# Green superlubricity of Nitinol 60 alloy against steel in presence of castor oil

**DOI:** 10.1038/srep29992

**Published:** 2016-07-21

**Authors:** Qunfeng Zeng, Guangneng Dong, Jean Michel Martin

**Affiliations:** 1 Key Laboratory of Education Ministry for Modern Design and Rotor-Bearing System, Xi’an Jiaotong University, Xi’an, 710049, China; 2 University of Lyon, LTDS, Ecole Centrale de Lyon, 69134, Ecully, France

## Abstract

In the present work, first, we show that sliding Nitinol 60 alloy against steel under castor oil lubrication exhibits a new case of superlubricity (coefficient of friction CoF ≪ 0.01). So far, CoF below 0.01 have never been achieved under boundary lubrication at high contact pressure and in presence of vegetable oil as a green lubricant. Next, it is demonstrated that superlubricity is controlled by tribochemical reactions, involving chemical degradation of castor oil and the formation of metal oxy-hydroxides. Finally, to explain these findings, we propose a novel superlubricity mechanism consisting of hexanoic acid molecules intercalated between nickel and iron oxy-hydroxide lamellar layers, a structure very similar to the one found in Fe-Ni batteries. We propose that superlubricity is achieved due to repulsive electrostatic forces acting between the intercalated metal oxy-hydroxide lamellar compounds. This system would be suitable for practical engineering applications in many fields including biotechnologies.

Nitinol 60 alloy is hard, highly corrosion resistant, and less dense than steel, non-galling and non-magnetic material and it exhibits excellent useful structural characteristics offering a broad combination of properties^1^. Based on these special characteristics, Nitinol 60 alloy has been considered as a promising candidate material for oil-lubricated rolling contact applications. DellaCorte *et al*. showed that Nitinol 60 alloy exhibits good tribological properties under synthetic hydrocarbon oil lubrication^2^. Coefficient of friction (CoF) of Nitinol 60 alloy is about 0.06 at the initial stage and then CoF reaches 0.3, a value which is too high for roller bearing applications. Therefore, it is very necessary and urgent to find new lubricants to reduce friction of Nitinol 60 alloys. Our previous works have shown that the lubrication performances of Nitinol 60 alloy can be substantially improved by the appropriate lubricants^3^,^4^. With the world becoming more environmental conscious, the lubricant oil should be environment friendly due to the need of clean environment. The green lubricants are those that optimize energy efficiency and minimize wear in the machinery which has maximized service lifetimes in order to reduce the amount of lubricants^5^. Castor oil is a good vegetable oil and a potential source of environmentally favorable friendly lubricants because of a combination of biodegradability, renewability and excellent lubrication performance^6^. Therefore, castor oil was chosen as a green lubricant to reduce the friction and wear of Nitinol 60 alloy. The main objective of the present work is to investigate the tribochemistry of castor oil during sliding and discuss the possible superlubricity mechanism of Nitinol 60 alloy against steel under castor oil lubrication.

## Results

**Figure 1:**
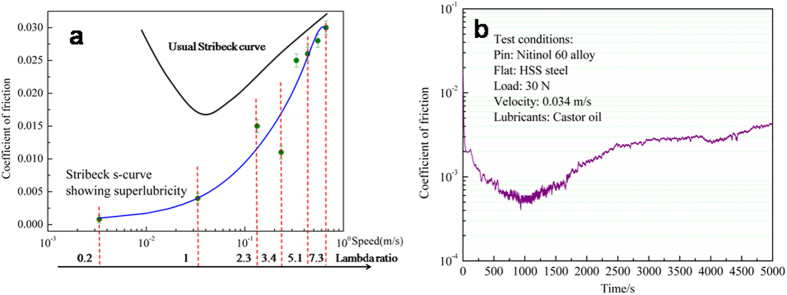
CoF of Nitinol 60 alloy against steel lubricated by castor oil at different speeds. (**a**) Stribeck curve usual shape and Stribeck curve with s-shape in the present work. (**b**) Evolution of CoF with Nitinol 60 alloy against sliding time. The steady state CoF at the load of 30 N and 3 mm/s shows vanishing of friction after 1000 seconds.

Figure 1a shows a so-called Stribeck curve representing the evolution of CoF versus the sliding speed in log scale and Lambda ratio under castor oil lubrication at ambient temperature and relative humidity of around 40%. Speeds increase from 3 mm/s to 0.67 m/s, the applied load is 30 N corresponding to a contact pressure of 280 MPa. Each value of CoF in the curve in Fig. 1a corresponds to a unidirectional test of 1000 seconds and the CoF value is recorded in the steady state at the end of the test. The bars for each CoF value in Fig. 1a represent the fluctuations around the average CoF value. For example, Fig. 1b represents the friction test at the speed of 3 mm/s, the lowest sliding speed investigated here. First, we observed the amazingly low values of CoF at low speed. The measuring range of the load sensor is from 2 N to 200 N. The sensitivity of the force tranducer is 10 mN, for a load of 30 N, this corresponds to a CoF of 3 × 10^−4^ that is the lowest CoF detectable with the tribometer. Second, it is very surprising to observe an inverted shape of this Stribeck curve compared with the typical Stribeck curves where friction increases sharply at the lowest sliding speeds, as shown in Fig. 1a. It is found that CoF increases from 0.0005 to 0.03 with the increase of sliding speeds. The lowest CoF is only about sixtieth of the highest value. The lambda ratio is also provided at different speeds in Fig. 1a. The lambda ratio (λ) is widely recognized as a standard dimensionless factor to decide which lubrication status is active. λ < 1 indicates the boundary lubrication conditions, which means that the asperities of pin and flat surfaces directly contact each other, resulting in high CoF. However, in the present case, CoF has a super low value of about 0.0005 under boundary lubrication. λ > 3 indicates EHL (elastohydrodynamic lubrication). CoF has almost the same value under high speeds in the standard Stribeck curve and in our s-shape Stribeck curve. Other value indicates the mixed lubrication, which means that both the boundary lubrication and EHL conditions exist in the contact region. CoF in the present case is lower than that in the standard Stribeck curve under the mixed lubrication. It is concluded from the experimental results that there is huge difference between standard Stribeck curve described in the literature and our s-shape Stribeck curve under castor oil lubrication.

In the following, we focus on the tribological properties of Nitinol 60 alloy/steel friction pair exhibiting the lowest CoF (as shown in Fig. 1b) at the load of 30 N (280 MPa contact pressure) and sliding speed of 3 mm/s. The evolution of CoF in the first seconds shows a sudden decrease from 0.03 to around 0.003 after only 20 seconds. Sometimes the CoF values below 0.001 were recorded although we are not sure that these values are significant considering the sensitivity and resolution of the force transducer. CoF decreases slowly to around 0.0005 after about 1000 seconds. Vanishing of the friction force (near friction-less sliding) is kept during 1600 seconds. Finally, CoF increases slightly to around 0.004 with sliding time duration. This experiment was conducted several times and although the evolution of CoF is not exactly the same in each test, the same level of CoF was recorded at each time. To determine the lubrication regime, we calculated the lambda ratio representing the value of the minimum oil film thickness divided by the composite roughness of the two contact surfaces. At 3 mm/s sliding speed, lambda ratio is ≈0.2 indicating that the tribotest is lubricated in the boundary regime, with the existence of solid contacts between the two metallic surfaces and a low contribution of the oil rheological properties.

**Figure 2:**
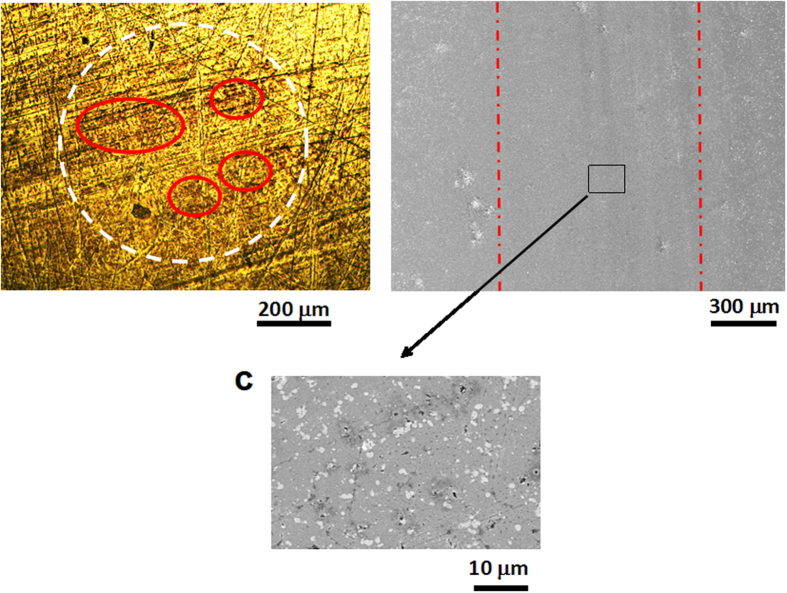
Worn surface images of the friction pair. (**a**) Optical image of pin. (**b**) SEM image of disc. (**c**) The worn surface of disc at higher magnification.

Figure 2 shows optical and SEM images of the friction pair worn surfaces obtained at the end of tribotest. It is seen that the contacting region of Nitinol 60 alloy pin is almost no wear after running for 5000 seconds (as shown in Fig. 2a) and the circular Hertzian zone is hardly visible. Only some darker areas on the apparent contact area of Nitinol 60 alloy pin can be observed and roughness of pin surface is apparently not affected during the friction test. SEM image of the wear scar on the steel disc (as shown in Fig. 2b) does not show any visible damage but only polishing inside the wear scar. A magnification of SEM image, Fig. 2c does not show any specific scratch in the direction of sliding. The white areas visible in Fig. 2c correspond to tungsten and molybdenum carbides grains present in high speed steel (HSS). From these observations, it can be deduced that only a colored very thin tribofilm is built on the pin surface and seems responsible of the amazing tribological properties of the present lubricating system.

**Figure 3:**
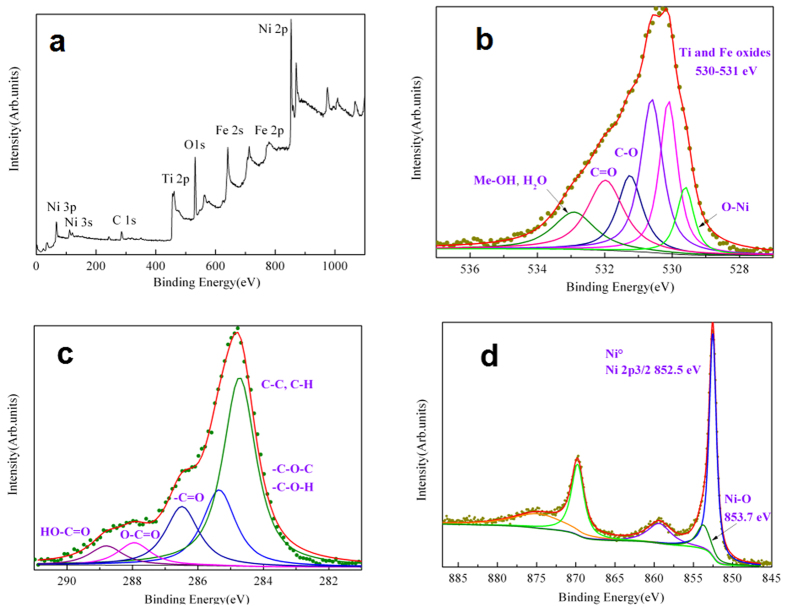
C 1s, O 1s and Ni 2p XPS spectra on the worn surface. (**a**) XPS general scan. (**b**) O 1s spectrum. (**c**) C 1 s spectrum. (**d**) Ni 2p spectrum.

To investigate the friction mechanism of Nitinol 60 alloy/steel friction pair under castor oil lubrication in detail, it is necessary to determine which kind of thin tribofilm is present on the worn surface of the friction pair. Therefore, worn surfaces of the friction pair were analyzed by Energy Dispersive X-ray Spectroscopy (EDS) and X-ray photoelectron spectroscopy (XPS). Figure 3a shows an overview of the XPS spectrum obtained inside the Nitinol alloy pin contact zone, showing clearly the Fe 2s and 2p peaks and the relatively low intensity of the C 1s peak. Semi-quantitative XPS analysis on the Nitinol alloy pin (see Table 1) shows that Ti, Ni, O and C elements are present in the tribofilm as expected. However, iron is also detected in a significant amount (11 at. %). The presence of iron in the Nitinol 60 alloy pin wear scar, certainly transferred from the steel disc, clearly confirms the existence of the boundary lubrication regime that was predicted by calculations and gives evidence that intimate contacts between metals have taken place during lubrication test. In addition, in the EDS analysis (as shown in Table 2), no iron is detected and this shows that iron detected by XPS is located on the top surface of the pin, in the tribofilm and not in the bulk. It is suggested that carbon and oxygen are derived from both castor oil and air meaning that tribochemistry plays a significant role in the formation of the thin tribofilm.

To understand the tribochemistry mechanism, the detailed analysis of deconvolated XPS spectra of the contact surface will provide clearly the evidence that the worn surface of the friction system was chemically modified with castor oil and oxygen. Figure 3c shows the high resolution XPS C 1s photopeak with different chemical states that can be detected at 284.8 eV, 286.3 eV, 287.8 eV and 288.8 eV. These species are characteristic of the presence of carbon in aliphatic chains (C-C and C-H) and oxidized carbon chemical groups like ether (-C-O-), hydroxyl (-COH), carbonyl (-C = O) and ester/acid C(O)OH, respectively^7^. Considering the chemical formula of castor oil, it is obvious that the original molecules have undergone chemical degradations since no carbonyl and acid functions are present in castor oil. O1s XPS peak shows also different contributions (as shown in Fig. 3b). The characteristic peak at 529.6 eV BE is generally attributed to metal oxides (NiO and FeO_x_) but other metal oxides like TiO_x_ are also present in O1s spectrum between 530 and 531 eV. The peaks in the region 531–532 eV correspond to O-C and O = C bonds from ether or carboxylic acid or esters but they can also correspond to metal hydroxides^8^. Ti 2p photopeak at 461 eV and Fe 2p at 713 eV are easier to identify and are characteristic of metal oxides species, in agreement with the O 1s spectrum.

The XPS peak of Ni 2p at 852.5 eV in the tribofilm (as shown in Fig. 3d) is strong (about 19at.%,). Biensenger’s XPS work gives very detailed information on Ni chemical states by using accurate reference nickel-based compounds^9^. By comparing our Ni 2p spectrum with Biensinger’s data, we can conclude that a major part of nickel is metallic and the rest is nickel oxide and/or hydroxide. The measured contents of Ni, NiO and others compounds including nickel hydroxide are respectively 51 at.%, 24 at.% and 25 at.% according to semi-quantitative analysis. The existence of NiO is also confirmed by the presence of O1s peak at 529.6 eV in Fig. 2b, which is characteristic of NiO. Nickel hydroxide is also present in the Ni 2p XPS spectrum and this is also confirmed by the presence of O1s peak at 532.9 eV. Usually, the metal hydroxides stand above the metal oxides. Nickel may be subject to oxidation by O_2_ on the pin surface in ambient air. On the basis of the tribological results and XPS analyses, nickel is oxidized to nickel hydroxide on the pin surface by oxygen and water vapor present in ambient air *via* the following reaction, as shown in equation (1).


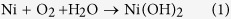


Interestingly, nickel hydroxide has a lamellar structure. Ni^2+^ can itself be oxidized to Ni^3+^. Nickel hydroxide can further be oxidized by oxygen to produce nickel oxy-hydroxide^10^, as shown in equation (2).


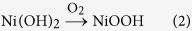


**Figure 4:**
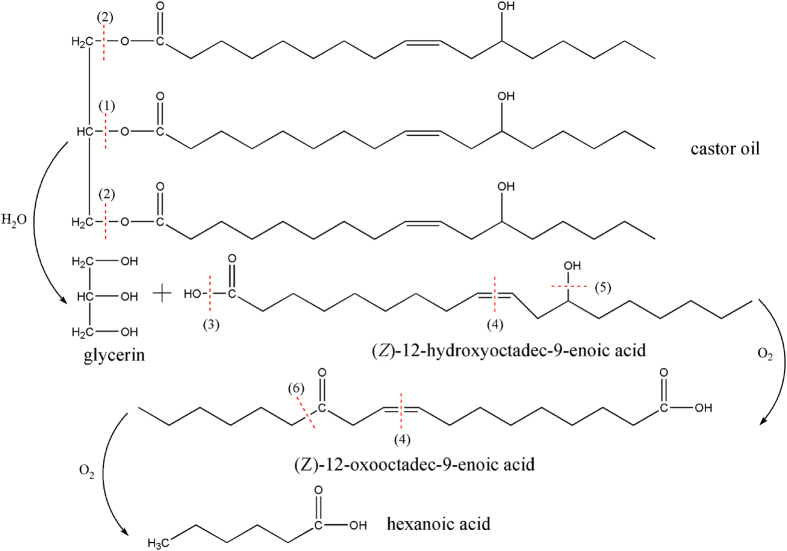
General radical reaction pathways for castor oil tribochemistry.

In the light of XPS analyses obtained here, the oxidation of castor oil is due to the presence of ester group, double bonds and hydroxyl group in castor oil molecules and this offers high level of reactivity with oxygen, especially when it is placed in friction contact with humid air under high friction heating and pressure simultaneously. The oxidation mechanism of castor oil can be explained as following:

1. Adsorption of castor oil molecules on the metallic surfaces. The carboxylic group of the fatty acid is certainly chemisorbed to the metal surface.
2. Hydrolysis of castor oil inside contact region under the combined effects of pressure, heat and shear. There is always a little water present even in high purity castor oil and that can also absorb water from humid ambient air. Therefore, the hydrolysis reaction of castor oil is unavoidable especially under the friction heating and high pressure simultaneously. At first, triacylglycerol group is decomposed into diacylglycerol and ricinoleate in castor oil. In the following hydrolysis, triacylglycerol group is decomposed into glycerol and ricinoleate.
3. Oxidation reactions. There are carboxylic group, double bond and hydroxyl group in ricinoleate. The electron attracting properties of the neighboring hydroxyl group, in addition to the field effect of carboxyl group, thus double bond increases the chemical reactive behavior of hydroxyl group. Therefore, hydroxyl group can be oxidized into carbonyl group in ricinoleate. The presence of nascent metallic nickel can strongly enhance the oxidation reaction of castor oil. The stability of carbonyl group is lower than that of C = C bond; therefore, C = C bond can be oxidized and cleave C = C bond in ricinoleate due to the friction heating and high pressure simultaneously. Oxidative cleavage of C = C bond to carbonyl compounds is an essential operation in organic synthesis^11^. The C = C bonds can be cleaved firstly and oxidized to carboxylic acid ultimately because there is a hydrogen atom on the alpha carbon linked C = C bonds^12^. Therefore, ricinoleate can be degraded into hexanoic acid (CH_3_(CH_2_)_4_COOH). Our XPS spectrum of carbon on the pin worn surface (Fig. 3c) is in good agreement with the formation of ethers (C-O), hydroxyl (C-OH), carbonyl (C = O) and acid C(O)OH. Therefore, it is concluded that castor oil may be degraded into hexanoic acid CH_3_(CH_2_)_4_COOH as the most stable degradation compound of castor oil during the running-in progress. Figure 4 is a schematic representation of general radical reactions and the evolutionary process of castor oil in detail.

**Figure 5:**
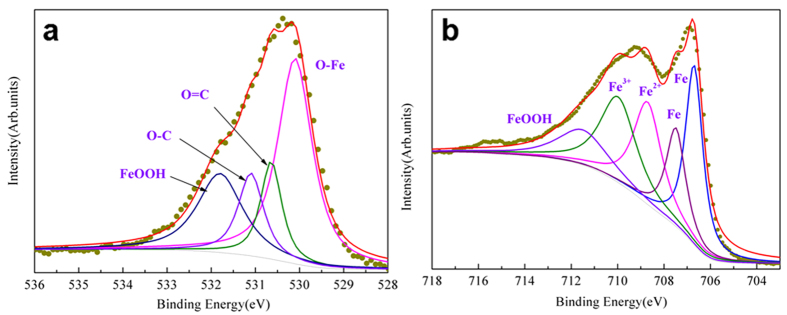
O 1s and Fe 2p XPS spectra on the disc worn surface. (**a**) O 1s spectrum (**b**) Fe 2p spectrum.

Figure 5 shows resolved XPS spectra of iron and oxygen obtained on the worn surface of the steel disc. No nickel is found on the steel disc surface and the thickness of tribofilm is only a few nanometers thick because the metal iron is easily detected underneath the oxide contribution (Fig. 5b). It is clear that some oxide/hydroxide species are present in the tribofilm. The peaks in the region 530–531 eV correspond to O-C and O = C bonds originating from ether or carboxylic acid or esters and O-Fe as shown in Fig. 5a. O 1s photopeak at 531.8 eV and Fe 2p at 711.5 eV are easier to identify and are characteristic of FeOOH.

From the above XPS analysis, it is inferred that there are NiOOH on the Nitinol pin worn surface, FeOOH on the disc worn surface and hexanoic acid, which may be coordinated with NiOOH and FeOOH. These results will provide the first experimental evidence to build the superlubricity model.

## Discussion

Superlubricity is defined as a sliding regime in which friction or wear resistance to sliding almost vanishes. Theoretically, superlubricity is the realization of zero friction force. But in practice, due to the limited precision of measurement and other factors, it is considered that when CoF is less than 0.01. Due to superlow friction, it has attracted increasing interest in recent years from both the researches and industrial communities. Superlubricity is divided in two mechanisms depending on the use of solid or liquid lubricants^13^,^14^,^15^,^16^,^17^. The superlubricity mechanism using solid lubricants such as diamond like carbon^18^ and molybdenum disulfide^19^ is very different from that of liquid lubricants such as polymer brushes and ceramic materials with water^20^,^21^,^22^, steel with glycerol solution or polyhydric alcohol^23^ and phosphoric acid^24^. Due to the differences in the contact conditions and friction pair material, there are lots of the superlubricity models to explain superlow friction in different lubrication systems^13^,^14^,^15^,^16^,^17^,^18^,^19^,^20^,^21^,^22^,^23^,^24^. Although it is found that hydrogen or OH is necessary to response superlubricity, however, these superlubricity models cannot be applied to explain our superlubricity phenomena totally. In our cases, the superlubricity of castor oil and Nitinol 60 alloy/steel lubrication system seems different from above mentioned mechanisms. Therefore, it is necessary that we propose a new superlubricity mechanism model coupling the mechanisms of solid and liquid superlubricity to explain the superlubricity behavior of this lubrication system under castor oil lubrication.

**Figure 6:**
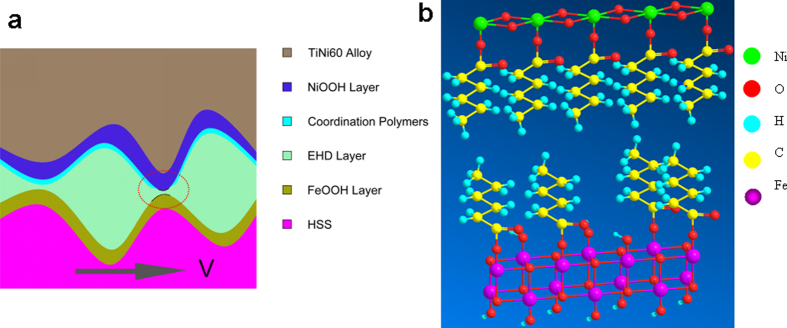
Schematic illustrations of partially di-hydrated hydrogen of the friction pair in lubrication model and molecular schematic presentation of the coordination layer and metal oxy-hydroxide. (**a**) The lubrication diagrammatic sketch of the friction pair under castor oil lubrication; (**b**) The hexanoic acid bonded metal oxy-hydroxide and fragment of a general intercalation NiOOH and FeOOH structure displaying the repulsive electrostatic forces that lead to slid easily between the contacting asperities.

Generally, there are two kinds of TiNi alloy including near-equiatomic TiNi alloy and Ni-rich alloy like Nitinol 60 alloy. Near-equiatomic TiNi alloy exhibits low hardness and superelastic, which results in high friction although high wear-resistance. However, it was found that Nitinol 60 alloy exhibits super low friction behavior (CoF below 0.01)^3^. The atomic radius (1.24 Å) of nickel atoms is lower than that of the titanium atoms (1.45 Å), which means the activation energy of nickel is lower than that of titanium. The nickel atoms can migrate and aggregate provoking an enrichment of the pin surface under external energy. Therefore, due to low activation energy of nickel, Nitinol 60 alloy is easily oxidized by oxygen in humid ambient air. At the initial stage, nickel is oxidized by oxygen and water molecular to form nickel oxide, and further oxidized by oxygen to nickel oxy-hydroxide by exposure to air under high friction heating and pressure during the friction and wear test. We remind here that the friction pair is lubricated by a meniscus of castor oil only and that humid air can easily reach with surface. From Ni 2p spectra results, after tribotest, nickel is mainly metal and nickel oxide/hydroxide at the top, Considering these observations, it seems that the chemical reduction of NiOOH gives rise to different forms of Ni(OH)_2_ that, in turn upon re-oxidation, generate largely NiOOH phases. Iron may experience almost the same oxidation procedure. In ambient air, FeOOH films will naturally grow on the iron surface^25^. As a result, a characteristic friction layer forms on both friction contact surfaces, and this friction layer determines the tribological properties of the lubrication system. The high content of poly-unsaturated fatty acids in castor oil makes it more prone to oxidative polymerization and dehydration-oxidation reactions between the fatty acid and iron oxide take place. At the same time, the pin and disc surfaces are also covered by fatty acids from castor oil. Therefore, the friction is mainly assumed to occur between asperities on OH-terminated surfaces, that makes CoF a little higher at initial stage. The friction heating increases with sliding time, castor oil is further oxidized to hexanoic acid and nickel oxide is further oxidized by oxygen to nickel oxy-hydroxide with lamellar structure providing weak interaction. In organic acids, surprisingly, the hexyl derivatives are air-stable over a 70–400 °C range, whereas other derivatives are stable only over a 30–300 °C range^26^. Therefore, the hexyl compound is the most stable. Bolln *et al*. observed that long-chain compounds exhibit odd-even effects, where the even-chain materials melted at temperatures that are 5–10 °C higher than adjacent odd-chain compounds^27^. It is reasonable to assume that hexanoic acid will eventually be considered as the most stable degradation compound for castor oil during the running-in progress. Eventually, hexanoic acid is intercalated by forming coordinate bonds with nickel oxy-hydroxide and iron oxy-hydroxide, which means that the repulsion force is increased. According to these experimental results and other researchers’ studies, a new superlubricity model is proposed. Figure 6 is a picture showing schematically the intercalation of hexanoic acid between nickel oxy-hydroxide and iron oxy-hydroxide. The dipole-dipole effects that form an interfacial Coulomb repulsion force also make a contribution to super low friction. Among all tribochemistry reaction products, there is also glycerol, which can form OH-terminated surfaces under high friction heating and pressure at the contact surface of the friction pair. Alternatively, superlubricity of Nitinol 60 alloy is also attributed to easy glide on triboformed OH-terminated surface.

This lubrication model is well supported by our analytical XPS results. NiO and Ni(OH)_2_ were observed by XPS in the present case even if they are not in large amounts probably because of solvent cleaning before analysis. Ni 2p peak at 853.7 eV and 874.7 eV and O 1 s peak at 529.6 eV and 532.9 eV are consistent with nickel oxide and nickel hydroxide or oxyhydroxide respectively after the tribotest according to XPS measurement. Ni 2p XPS peak of hydroxide can be well distinguished from the NiO one. The hexanoic acid in the tribochemical reaction products of castor oil during sliding has then the possibility to bond nickel oxy-hydroxide. The lone pair electron of oxygen in hydroxyl group of hexanoic acid is introduced to the coordination bond with lone pair electron of nickel in nickel oxy-hydroxide. The carboxylic group of hexanoic acid reacting with hydroxyl group of iron oxy-hydroxide layer may form the repulsive force between metal oxy-hydroxide layers. As a result, hexanoic acid could park in an interpenetrating form, forming to a coordination layer. The expansion of organic acid coordinational nickel oxy-hydroxide is originated from the increase of organic acid content, whereas the basic structural framework and thickness of host slabs seem identical.

This lamellar structure through the coordination and chemical reaction can explain super low friction under green lubricating oil lubrication. At the initial stage, friction occurs between castor oil and the contact surface of the friction pair under boundary lubrication, there is chemical reaction between castor oil and the friction surface, forming NiOOH and FeOOH, which makes low friction, and CoF decreases slowly with the increase of sliding time. At the same time, castor oil is oxidized, and breakdown of castor oil molecules during tribochemical process could result in the formation of fatty acids that can further react with iron from steel. The hydrocarbon chain of the fatty acid provides an excellent molecular barrier while the polar group coordinates with iron to form a protective film on the steel surface. The chemical reaction products of castor oil are adsorbed to the friction interface, forming coordinate bond and generating dehydration reaction between iron, nickel and organic reactant. Therefore, superlubricity is achieved under NiOOH layer, coordination layer, EHL layer and FeOOH layer simultaneously. With the increase of sliding time, hexanoic acid can be oxidized by oxygen under high friction heating and pressure, even oxidized to carbon dioxide and water. Nickel oxide can further also be oxidized by oxygen, as shown in equation (3).


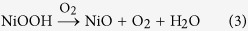


These changes cause a little higher CoF during the friction test.

**Figure 7:**
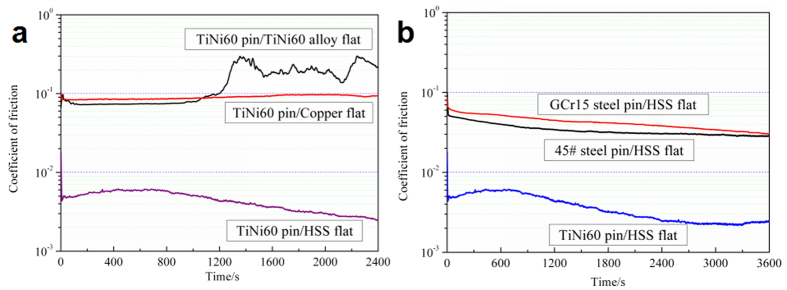
CoF of different friction pairs lubricated by castor oil at ambient temperature. (**a**) Nitinol 60 alloy pin; (**b**) HSS steel flat.

The superlubricity originating from Nitinol 60 alloy and steel under castor oil lubrication has been obtained. It is found that the achievement of superlubricity is related to two important conditions. The first is the triboformed OH-terminated surface with nickel and iron oxy-hydroxide on the pin and steel in the contact region, respectively. The second is the positively charged surfaces induced by the hexanoic acid via the coordination and chemical reaction. According to this superlubricity mechanism, it could be inferred that if there is a lubricate system meeting these two conditions simultaneously then superlubricity would appear. Therefore, the influence of the friction pair material on superlubricity was discussed to reveal the key factors in achieving superlubricity because the influence of lubricating oil has already been investigated by the previous work^4^. To verify the importance of Nitinol 60 alloy/steel friction pair material in the lubrication model and investigate the interaction between castor oil and friction contact surface, we investigated few friction pairs under the same friction conditions. Figure 7 shows CoF of different friction pairs under castor oil lubrication. CoF of Nitinol 60 alloy/Nitinol 60 alloy friction pair is relative stable at the initial stage, as shown in Fig. 7(a), and CoF increases to about 0.3 after around 1200 seconds and fluctuates in the subsequence test. No super low friction is observed in this case. CoF of Nitinol 60 alloy/copper friction pair is relative stable along the sliding time but it does not reach superlow values. CoF of GCr15 steel/HSS and Nitinol 60 alloy/HSS friction pairs are relatively low comparing Nitinol 60 alloy/copper friction pair. These experimental results show that all the friction pairs could achieve low CoF below 0.1 in the steady state. However, only Nitinol 60 alloy/HSS friction pair exhibits superlow friction below 0.01, as shown in Fig. 7(a). It means that the material of the friction pair would be appropriate to obtain low friction due to the tribochemical reactions between castor oil and friction contact surface. For Nitinol 60 alloy/Nitinol 60 alloy friction pair, the OH-terminated surface maybe formed since Nitinol 60 alloy surface may be chemically reacted with oxygen and the chemical products of castor oil during the friction tests, but the hardness of Nitinol 60 alloy (around 420 HV) is too low to support high contact pressure and at the initial stage the OH-terminated surface would be destroyed, which results in relatively high CoF. It is almost the same situation for the Nitinol 60 alloy/copper friction pair due to low hardness of copper. For the GCr15 steel/HSS friction pair and 45^#^ steel/HSS friction pair, the hardness of the friction pair (around 800 HV) is relatively high. Under oil lubrication condition, the adhesion and plastic deformation of steel/steel contact surface asperities in the contact area seems to be the dominant mechanisms which determined the friction behavior of the self-mated steel friction pairs, therefore, it is difficult to achieve superlow friction of steel/steel friction pair even under oil lubrication condition because there always exists abrasive wear and the activated oxidation of the fresh steel surface created by abrasion in ambient air. To conclude, it is the unique combination of the presence of Ni in the pin, the presence of iron in steel flat and the presence of branched OH group in the backbone of the castor oil molecules that permit the intercalated Fe/Ni intercalated nanostructures to be formed and this provides ultimately ultralow friction. Interestingly, this kind of nanostructures is very similar to the one described in details in the case of Ni/Fe batteries^28^.

In summary, we achieved superlubricity (CoF in the 10^−3^ range and even less) for Nitinol 60 alloy sliding against steel under castor oil lubrication in the boundary regime, which means a novel way of probing superlubricity in practical use in many applications fields including biotechnologies. Other vegetable oils did not provide such superlubricity which means that the specific hydroxyl groups branched on the acid chain is necessary. It is obvious that the mechanisms to explain such low friction are very complicated from the chemical point of view. Based on the experiments and analysises, we build a new superlubricity model consisting of hexanoic acid molecules intercalated between nickel and iron oxy-hydroxide. This lamellar structure through the coordination and chemical reaction can explain superlow friction under green lubricating oils lubrication. Therefore, the superlubricity mechanism is attributed to the positively charged atomic planes formed by the hydrogen terminated carbon atoms of hexanoic acid derived from friction-induced oxidation of castor oil coordinated nickel and iron oxy-hydroxide and the triboformed OH-terminated surface with iron oxy-hydroxide. The tribological system is able to provide the strong repulsive electrostatic forces to achieve superlubricity behavior. This is beneficial to promote the energy efficiency, environment protection and has tremendous potential for applications in many industrial fields.

## Methods

### Friction measurements

Chemical composition and structure of Nitinol 60 alloy can be found in references 3. The tribotests of Nitinol 60 alloy/steel friction pair were carried out using a tribometer (UMT-2 from Bruker Corp.) with a pin-on-disc configuration, unidirectional sliding, under castor oil lubrication in ambient air at room temperature and relative humidity of around 40%. A hemispherical static pin made of Nitinol 60 alloy (surface roughness 0.2 μm) was slid against a disc made of high speed tool steel (HSS) with surface roughness value of 0.01 μm. The pin radius is 6 mm and height is 15 mm. A normal load is 30 N corresponds to a calculated maximum contact pressure of 280 MPa and sliding velocity can be varied from 3 mm/s to 0.67 m/s. Castor oil whose composition and viscosity is shown in our previous paper is provided in a minimal quantity as a meniscus (no reservoir), in order to minimize friction forces due to dragging liquid during the rotation movement. A calculation of central film thickness of castor oil in the interface gives a value of 0.15 μm for 3 mm/s (details can be found in a previous publication^3^). The lambda value (film thickness divided by composite roughness) is equal to 0.2 in agreement with boundary lubrication regime although EHL contribution in some part of the contact area cannot be completely excluded. The frictional data were recorded and displayed graphically by the software on the UMT-2 tribometer. After the tribotests, the worn surface of the friction pair was observed by optical microscope and scanning electron microscope (SEM) and analyzed by XPS measurements.

### XPS measurements

After the friction and wear tests, the samples were cleaned ultrasonically with acetone, and then the tribofilms on the contact surface of the Nitinol 60 alloy pin and the steel disc were investigated by XPS analysis. For XPS, non-monochromatized Mg Kα radiation as X-ray source (hν = 1253.6 eV) was used for photoelectron generation and characterization of the chemical composition of the friction pair. The focus area of the X-ray spot is about 50 μm, permitting XPS analysis inside most of the wear scar. The energy resolution is about 0.5 eV. Argon etching corresponds approximately to the removal of few-nanometers thick layer of materials from the worn surface. The obtained XPS data were normalized and then processed by the XPS software, using a peak-fitting routine with symmetrical Gaussian-Lorentzian functions. Special attention has been paid to the deconvolution of Fe 2p, C 1s, Ni 2p and O 1s photopeaks. For the fit, a Shirley-type background was subtracted from the photoelectron spectra.

## Additional Information

**How to cite this article**: Zeng, Q. *et al*. Green superlubricity of Nitinol 60 alloy against steel in presence of castor oil. *Sci. Rep.* **6**, 29992; doi: 10.1038/srep29992 (2016).

## Figures and Tables

**Table 1:** XPS results of Nitinol pin worn surface.

**Element**	**C 1s**	**Fe 2p**	**Ni 2p**	**O 1s**	**Ti 2p**
Content (at.%)	15.74	11.56	19.00	35.88	17.82

**Table 2:** EDS analysis results of Nitinol pin worn surface.

**Element**	**C K**	**Ni K**	**Ti K**
Content (wt.%)	0.8	55.4	43.8
